# Effects of SARS-CoV-2 Infection During Late Pregnancy on Early Childhood Development: A Prospective Cohort Study

**DOI:** 10.3389/fped.2021.750012

**Published:** 2021-11-23

**Authors:** Tianchen Wu, Lian Chen, Yuanyuan Wang, Huifeng Shi, Jieqiong Niu, Xiaohan Yin, Mengshi Li, Chang Tan, Hai Jiang, Danni Zheng, Yuan Wei, Yangyu Zhao, Xiaoli Wang, Jie Qiao

**Affiliations:** ^1^Department of Maternal and Child Health, School of Public Health, Peking University, Beijing, China; ^2^Department of Obstetrics and Gynecology, Peking University Third Hospital, Beijing, China; ^3^National Clinical Research Center for Obstetrical and Gynecology, Beijing, China; ^4^National Center for Healthcare Quality Management in Obstetrics, Beijing, China; ^5^Key Laboratory of Reproductive Health, National Health Commission, Beijing, China

**Keywords:** COVID-19, SASRS-CoV-2, pregnancy, early childhood development, mother-infant separation, mediation model

## Abstract

**Background:** There is little direct or indirect evidence of the effects of severe acute respiratory syndrome coronavirus 2 (SARS-CoV-2) infection during pregnancy on early childhood development.

**Methods:** We conducted a prospective, observational cohort study in China from May 1 to October 31, 2020, that enrolled 135 mother-infant dyads: 57 dyads in the infection cohort and 78 in the non-infection cohort. Among all infants, 14.0% were preterm birth in the infection cohort and 6.4% in the non-infection cohort. Participants were followed by telephone interviews to collect demographic characteristics, medical records of coronavirus disease 2019, breastfeeding data, and early childhood development was assessed by the Age and Stage Questionnaire (ASQ-3) and Age and Stage Questionnaire Social-Emotional (ASQ:SE-2) Chinese versions at 3 months after childbirth. We used multivariable Poisson regression models to estimate the relative risk (RR) of SARS-CoV-2 infection. Multivariable linear regression models and a mediation model were used to test the direct and indirect associations between SARS-CoV-2 infection and the ASQ-3 score. This study was approved by the Peking University Third Hospital Medical Science Research Ethics Committee (No. IRB00006761-M2020127).

**Results:** In the infection cohort, 13.6% of the children showed social–emotional developmental delay, and 13.5% showed overall developmental delay. The corresponding rates in the non-infection cohort were 23.4 and 8.1%. Compared with the non-infection cohort, SARS-CoV-2 infection during pregnancy did not increase the risk of social-emotional (RR = 0.87, 95% CI: 0.51–1.49) or overall (RR = 1.02, 95% CI: 0.60–1.73) developmental delay. The mediation model showed that SARS-CoV-2 infection indirectly affected the ASQ-3 score by increasing the length of mother–infant separation.

**Conclusions:** SARS-CoV-2 during late pregnancy did not increase the risk of developmental delay of the offspring 3 months after delivery. However, SARS-CoV-2 may have indirect effects on early childhood development by increasing mother-infant separation.

## Introduction

The coronavirus disease 2019 (COVID-19) pandemic is the most serious global public health crisis of the 21st century. As of July 29, 2021, more than 190 million people worldwide have been infected with severe acute respiratory syndrome coronavirus 2 (SARS-CoV-2) (1). Coronavirus infection affects pregnant women more than the general population, and infected pregnant women are more likely to need intensive care treatment than non-pregnant women (2, 3). The most common reported adverse perinatal outcomes related to COVID-19 are preterm birth and cesarean delivery (3–5).

Another concern with COVID-19 infection during pregnancy is that of the potential effects on early childhood development (6), which might occur *via* two possible pathways. The first is the pathophysiological effects of SARS-CoV-2 infection. Although, there is insufficient evidence of vertical SARS-CoV-2 infection transmission (7), mothers infected during pregnancy have higher interleukin-6 (IL-6) levels than non-pregnant women (8). IL-6 is an indicator of maternal systemic inflammation that is potentially related to infant brain development (9) and inversely associated with offspring cognition at 12 months of age (10). The second pathway is mother–infant separation caused by hospitalization. In the early phase of the pandemic, due to insufficient understanding of the transmission dynamics of COVID-19, and to treat infected pregnant women promptly and protect their newborns, mothers were separated from their infants for long periods (11). Mother–infant separation in early childhood has adverse effects on child development (12). Unfortunately, there is no evidence of whether SARS-CoV-2 infection during pregnancy increases the risk of neurodevelopmental delay in early childhood.

Our team has reported the clinical characteristics of pregnant women infected with SARS-CoV-2 (in the third trimester, mainly in the late trimester) and assessed the early development of their children (4, 13) using the National Epidemic Reporting System (NERS) of the National Health Commission of China. In this study, we used the previous data as an infection cohort, and established a non-infection cohort in Wuhan, China, recruiting pregnant women and their infants who had never been infected with SARS-CoV-2, to compare the early childhood development of the two cohorts and explore the potential secondary effects of COVID-19 pandemic on early childhood development.

## Materials and Methods

### Study Design and Participants

**Figure 1:**
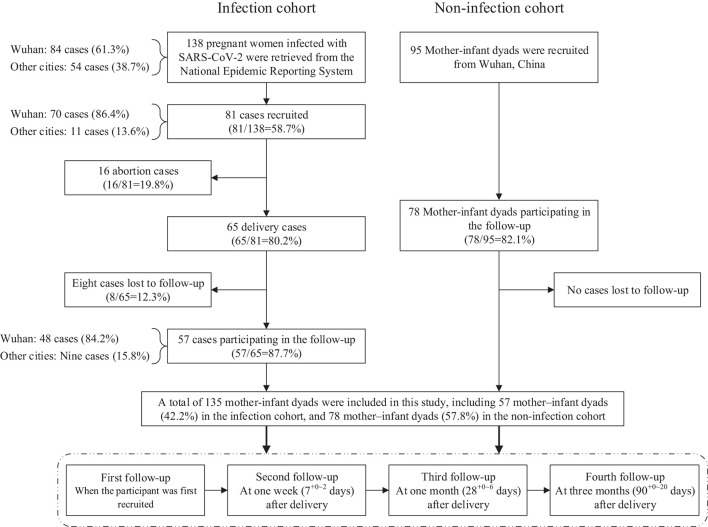
Flowchart of the prospective cohort study.

This prospective, observational cohort study was conducted from May 1 to October 31, 2020. It established an infection cohort who were followed from May 1 to July 1, 2020, and a non-infected cohort who were followed from July 7 to October 31, 2020. The infection cohort were retrieved from the NERS until April 30, 2020. The inclusion criteria of the infection cohort were (1) a confirmed case of COVID-19 defined as a suspected case (a person who had both the epidemiological history and clinical manifestations) with a positive result on high-throughput sequencing or real-time reverse transcription polymerase chain reaction (RT-PCR) assay of nasal and pharyngeal swab specimens (14), (2) a pregnant woman diagnosed with COVID-19, (3) the onset of COVID-19 was at any week of pregnancy, and (4) informed consent was obtained. Of the 138 cases retrieved from the NERS, 81 met the inclusion criteria, including 65 deliveries and 16 abortions. We included only the 65 deliveries of whom 57 (87.7%) of the women completed all follow-up procedures (13) (Figure 1).

The non-infection cohort were recruited through an online assistance and consultation platform for pregnant women in Wuhan initiated by doctors during the COVID-19 pandemic. The inclusion criteria of the non-infection cohort were (1) a pregnant woman not diagnosed with COVID-19, (2) not a close contact or suspected case, or not quarantined, (3) a live birth without neonatal death, and (4) informed consent was obtained. Ninety-five cases met the inclusion criteria and 78 (82.1%) completed all of the follow-up procedures (Figure 1). In the recruitment of participants, we did not limit the gestational age at delivery in both infection cohort and non-infection cohort. This study was approved by the Peking University Third Hospital Medical Science Research Ethics Committee (No. IRB00006761-M2020127).

### Follow-Up Procedures

For all participants in both cohorts, we conducted a follow-up at four time points. (1) The first was to obtain informed consent when the participant was recruited, and we collected the diagnosis, treatment, and outcomes of COVID-19 from medical records. (2) One week (7^+0~2^ days) after delivery, we collected data on hospitalized delivery, screening results of SARS-CoV-2 for maternal and neonatal specimens (RT-PCR assay of neonatal throat swab, cord blood, amniotic fluid, breast milk, meconium, or placenta, and IgG and IgM in neonatal serum), postpartum care from medical records; the duration of quarantine, mother–infant separation, and neonatal feeding were collected by telephone interviews. (3) At 1 month (28^+0~6^ days), the durations of quarantine and mother–infant separation, and neonatal feeding were obtained by telephone interviews. (4) At 3 months (90^+0~20^ days), the durations of quarantine and mother–infant separation, and neonatal feeding were obtained by telephone interviews, and early childhood development was assessed by sending an online questionnaire to the mobile phones of the mothers. The breastfeeding data collected in our study referred to both exclusively and partial breastfeeding; we did not distinguish them. Mother–infant separation included two scenarios, the first followed national policy (11) requiring neonates delivered by infected pregnant women to be observed in an isolated observation ward for at least 14 days. The second was that after the termination of medical quarantine, some mothers chose to continue home quarantine, which extended the mother–infant separation time.

### Early Childhood Development Assessment

Early childhood development was assessed using the Chinese versions of the Age and Stage Questionnaire (ASQ-3) and Age and Stage Questionnaire Social–Emotional (ASQ:SE-2). All infants were assessed at 3 months after childbirth. ASQ-3 consists of five domains: communication, gross motor, fine motor, problem solving, and personal–social. ASQ:SE-2 was used to assess social–emotional development. In this study, the mothers of the infants completed both ASQ-3 and ASQ:SE-2. A child whose ASQ-3 score was lower than the Chinese norm indicated developmental delay in that domain, and developmental delay in any ASQ-3 domain indicated overall developmental delay (15). A total ASQ:SE-2 score higher than the Chinese norm indicated social–emotional developmental delay (16).

### Statistical Analyses

Continuous variables were described as the median and interquartile range (IQR); the two cohorts were compared using the Wilcoxon signed-rank test. Categorical variables were described as the frequency and percent, and the two cohorts were compared using the Chi-square test or Fisher's exact test. We estimated the adjusted relative risk (RR) of SARS-CoV-2 infection on developmental delay using multivariable Poisson regression models. The associations between SARS-CoV-2 infection and the ASQ-3 and ASQ:SE-2 scores were tested using multivariable linear regression models. We adjusted for the length of mother–infant separation, low birthweight (yes vs. no), infant gender (boy vs. girl), preterm (yes vs. no), admitted to neonatal intensive care unit (NICU; yes vs. no), and breastfeeding at 3 months after delivery (yes vs. no) in the multivariable Poisson and linear regression models.

Based on the preliminary analysis, we hypothesized that the effects of SARS-CoV-2 infection on early childhood development may be mediated by mother–infant separation. Therefore, we fitted a mediation model to test the indirect effect. This mediation model included five paths: Path a represented the effect of SARS-CoV-2 infection on the length of mother–infant separation; Path b represented the effect of length of mother–infant separation on gross motor development; Path a × b (indirect effect) represented the mediating effect of SARS-CoV-2 infection on gross motor development mediated by length of mother–infant separation; Path c′ (direct effect) represented the remaining effect of SARS-CoV-2 infection on gross motor development that could not be explained by the mediating effect in this model; and Path c (total effect) represented the total effect of SARS-CoV-2 infection on gross motor development.

Descriptive analyses and multivariable analyses were implemented using SAS ver. 9.4 (SAS Institute, Cary, NC, USA). The mediation model was implemented using the mediation package PROCESS ver. 3.5 by Andrew F. Hayes for SPSS (17). All statistical tests were two-tailed, and *p* < 0.05 was considered statistically significant.

## Results

### Demographic Characteristics and Main Outcomes

Table 1 summarizes the demographic characteristics, perinatal conditions, and early childhood development. The median age of the mothers was significantly higher in the infection cohort. A significantly lower proportion of mothers in the infection cohort had a bachelor's degree or above. The proportions of low birthweight, congenital malformation, fetal distress, neonatal asphyxia, admitted to NICU, and preterm birth did not differ significantly between the two cohorts.

**Table 1:** Demographic characteristics, perinatal outcomes, and early childhood development between infection cohort and non-infection cohort.

**Characteristics**	**Infection cohort (*n* = 57)**	**Non-infection cohort (*n* = 78)**	***p*-value**
**Maternal characteristics**			
Maternal age, median (IQR)	31.2 (28.3, 34.4)	29.0 (28.0, 31.0)	0.004^b^
Maternal education (bachelor or above), *n* (%)	41 (71.9%)	73 (93.6%)	<0.001^c^
Working mothers, *n* (%)	30 (52.6%)	47 (60.3%)	0.377^c^
Maternal complication, *n* (%)	13 (22.8%)	20 (25.6%)	0.705^c^
Gestational age at diagnosis of COVID-19, median (IQR)	37.0 (34.2, 38.6)	–	–
**Perinatal outcomes**			
Low birthweight (<2,500 g), *n* (%)	4 (7.0%)	3 (3.8%)	0.452^d^
Birthweight, median (IQR)	3,100 (2,800, 3,450)	3,270 (3,030, 3,600)	0.034^b^
Infant gender (girls), *n* (%)	29 (50.9%)	40 (51.3%)	0.963^c^
Congenital malformation, *n* (%)	1 (1.8%)	2 (2.6%)	1.000^d^
Fetal distress, *n* (%)	1 (1.8%)	5 (6.4%)	0.400^d^
Neonatal asphyxia, *n* (%)	1 (1.8%)	3 (3.9%)	0.638^d^
Admitted to NICU, *n* (%)	11 (19.3%)	8 (10.3%)	0.136^c^
Preterm birth (<37 weeks), *n* (%)	8 (14.0%)	5 (6.4%)	0.138^c^
**Mother-infant separation days, median (IQR)**	37.5 (21.0, 52.5)	0 (0, 0)	<0.001^b^
**Breastfeeding**			
At 1 week after delivery, *n* (%)	5 (8.8%)	75 (96.2%)	<0.001^c^
At 1 month after delivery, *n* (%)	11 (19.3%)	78 (100.0%)	<0.001^c^
At 3 months after delivery, *n* (%)	21 (36.8%)	78 (100.0%)	<0.001^c^
**Early childhood development^a^**			
Social–emotional delay, *n* (%)	6 (13.6%)	15 (23.4%)	0.206^c^
Score of social emotion, median (IQR)	25 (10, 35)	25 (10, 40)	0.732^b^
Overall developmental delay, *n* (%)	7 (13.5%)	5 (8.1%)	0.350^c^
Communication delay, *n* (%)	0 (0.0%)	2 (3.2%)	0.500^d^
Score of communication, median (IQR)	50 (40, 60)	50 (40, 55)	0.507^b^
Gross motor delay, *n* (%)	0 (0.0%)	0 (0.0%)	–
Score of gross motor, median (IQR)	55 (45, 60)	60 (55, 60)	0.006^b^
Fine motor delay, *n* (%)	3 (5.8%)	0 (0.0%)	0.092^d^
Score of fine motor, median (IQR)	45 (40, 55)	55 (45, 60)	0.038^b^
Problem solving delay, *n* (%)	3 (5.8%)	3 (4.8%)	1.000^d^
Score of problem solving, median (IQR)	50 (40, 55)	55 (45, 60)	0.002^b^
Personal–social delay, *n* (%)	5 (9.6%)	2 (3.2%)	0.243^d^
Score of personal–social, median (IQR)	45 (40, 55)	50 (45, 55)	0.002^b^

*IQR, interquartile range; NICU, neonatal intensive care unit*.

a *In the infection cohort, 13 participants did not respond to ASQ:SE-2, five participants did not respond to ASQ-3; In the non-infection cohort, 14 participants did not respond to ASQ:SE-2, 16 participants did not respond to ASQ-3*.

b *Wilcoxon signed-rank test*.

c *Chi-square test*.

d *Fisher's exact test*.

In the infection cohort, four pregnant women (7.0%) were infected in the second trimester (19.5, IQR = 15.8–23.1 weeks) and the other 53 (93.0%) in the third trimester (37.4, IQR = 35.1–38.8 weeks). Clinically, 49 (86.0%) pregnant women were diagnosed as mild cases and eight (14.0%) as severe cases. Furthermore, we collected screening results of RT-PCR assay of neonatal throat swab (51 cases), cord blood (3 cases), amniotic fluid (2 cases), breast milk (12 cases), meconium (3 cases), and placenta (2 cases). A single neonate had a positive RT-PCR throat swab 36 h after delivery, but was negative in subsequent tests, as previously reported (13, 18). There were 17 cases having a testing of IgG and IgM in neonatal serum: eight cases (47.1%) with IgG (+), including three cases (17.6%) with IgM (+) (13).

From the date of birth, the separation time of mothers and infants in the infection cohort was significantly longer than that in the non-infection cohort. In the non-infection cohort, eight mothers were separated from their infants for a median of 19 (IQR = 7.0–36.0) days because their infants were admitted to the NICU. The proportions of breastfeeding (did not distinguish exclusively and partial breastfeeding) in the infection cohort at 1 week (8.8 vs. 96.2%), 1 month (19.3 vs. 100.0%), and 3 months (36.8 vs. 100.0%) were significantly lower than in the non-infection cohort. Social–emotional developmental delay was seen in 13.6% of the children in the infection cohort and 23.4% of those in the non-infection cohort (*p* > 0.05). Similarly, overall developmental delay was detected in 13.5% of the children in the infection cohort and 8.1% in the non-infection cohort; the difference was also not significant. The proportions with developmental delay in communication, fine motor, problem solving, and personal–social did not differ significantly between the two cohorts. No children were assessed as having gross motor developmental delay in either cohort. However, the gross motor, fine motor, problem solving, and personal–social ASQ-3 scores were significantly lower in the infection cohort.

### Association Between SARS-CoV-2 Infection and Early Childhood Development

After adjusting for potential confounders, we found that SARS-CoV-2 infection during pregnancy did not increase the risks of social–emotional (RR = 0.87, 95% CI: 0.51–1.49) and overall (RR = 1.02, 95% CI: 0.60–1.73, Table 2) developmental delay. SARS-CoV-2 infection was also not significantly associated with the ASQ-3 and ASQ:SE-2 scores. Although we found that mother–infant separation did not increase the risks of social–emotional (RR = 1.00, 95% CI: 0.99–1.01) and overall (RR = 1.00, 95% CI: 0.99–1.01, Table 2) developmental delay, multivariable linear regression models indicated that the length of mother–infant separation was significantly negatively correlated with the gross motor score (β = −0.09, 95% CI: −0.16 to −0.02).

**Table 2:** Association between SARS-CoV-2 infection and early childhood development.

**Early childhood development**	**RR (95%CI)^a^**	***p*-value**	**β (95%CI)^b^**	***p*-value**
**Social emotion**				
SARS-CoV-2 infection	0.87 (0.51, 1.49)	0.617	2.94 (−5.11, 11.00)	0.474
Mother–infant separation days	1.00 (0.99, 1.01)	0.906	−0.11 (−0.27, 0.04)	0.154
**Overall development^c^**				
SARS-CoV-2 infection	1.02 (0.60, 1.73)	0.951	–	–
Mother–infant separation days	1.00 (0.99, 1.01)	0.963	–	–
**Communication**				
SARS-CoV-2 infection	1.01 (0.58, 1.75)	0.968	0.76 (−4.62, 6.14)	0.782
Mother–infant separation days	1.00 (0.99, 1.01)	0.898	−0.06 (−0.17, 0.04)	0.252
**Gross motor^d^**				
SARS-CoV-2 infection	–	–	−0.57 (−4.05, 2.92)	0.751
Mother–infant separation days	–	–	−0.09 (−0.16, −0.02)	0.008
**Fine motor**				
SARS-CoV-2 infection	1.04 (0.60, 1.81)	0.887	−1.65 (−7.18, 3.88)	0.559
Mother–infant separation days	1.00 (0.99, 1.01)	0.889	−0.08 (−0.19, 0.03)	0.146
**Problem solving**				
SARS-CoV-2 infection	1.02 (0.60, 1.75)	0.935	−0.86 (−5.87, 4.14)	0.736
Mother–infant separation days	1.00 (0.99, 1.01)	0.970	−0.08 (−0.18, 0.02)	0.111
**Personal-social**				
SARS-CoV-2 infection	1.01 (0.59, 1.74)	0.973	−0.43 (−6.87, 6.02)	0.897
Mother–infant separation days	1.00 (0.99, 1.01)	0.993	−0.02 (−0.13, 0.12)	0.958

a *Adjusted relative ratio (RR) calculated by Poisson regressions, social–emotion developmental delay, and overall developmental delay were used as binary dependent variable in the Poisson regressions;*

b *coefficient of linear regressions, the scores of ASQ-3 and ASQ:SE-2 were used as dependent variables in the linear regressions;*

c *the overall developmental delay was defined as any domain of ASQ-3 that was assessed as developmental delay, which did not have a score and did not need to be considered in linear regressions;*

d *no children were assessed as gross motor delay in the infection cohort and the non-infection cohort, so gross motor was not considered in the Poisson regression. In both Poisson regressions and linear regressions, we adjusted maternal education (bachelor or above vs. high school or below), low birthweight (yes vs. no), infant gender (boy vs. girl), preterm (yes vs. no), admitted to NICU (yes vs. no), and breastfeeding at 3 months after birth (yes vs. no)*.

### Mediation Effect Between SARS-CoV-2 Infection and Early Childhood Development

**Figure 2:**
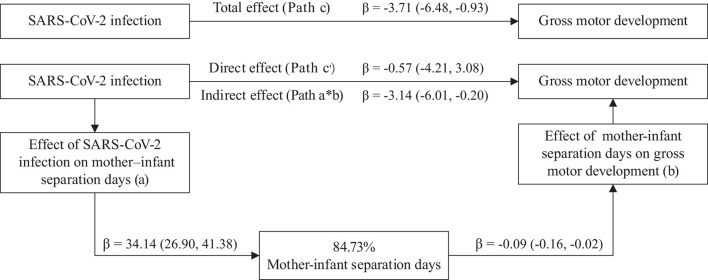
Mediation model of SARS–CoV−2 infection effect on gross motor development. Path a, the effect of SARS–CoV−2 infection on mediator. Path b, the effect of mediator on gross motor development. Path a*b (indirect effect), the mediated effect of SARS–CoV−2 infection on gross motor development by mother–infant separation days. Path c′ (direct effect), the remaining effect of SARS–CoV−2 infection on gross motor development extent not explained by the mediators included in the model. Path c (total effect), the total effect of SARS–CoV−2 infection on gross motor development. In the mediation model, we adjusted maternal education (bachelor or above vs. high school or below), low birthweight (yes vs. no), infant gender (boy vs. girl), preterm (yes vs. no), admitted to NICU (yes vs. no), and breastfeeding at 3 months after delivery (yes vs. no).

Table 3 and Figure 2 show the components of the mediation model. SARS-CoV-2 infection had significant total (Path c, *p* = 0.009) and indirect (Path a × b) effects on the gross motor development scores; the indirect effect and 84.73% of the total effect were mediated by the length of mother–infant separation. SARS-CoV-2 infection significantly increased the length of mother–infant separation (Path a, *p* < 0.001), which significantly affected the gross motor development score (Path b, *p* = 0.012). The remaining direct effect of SARS-CoV-2 infection on the gross motor development score was not significant (Path c′, *p* = 0.759).

**Table 3:** Mediation effect between SARS-CoV-2 infection and score of gross motor domain of ASQ-3.

**Mediation path**	**β (95%CI)**	***p*-value**	Proportion mediated (a*b/c)
**COVID-19 effect on mediator** (Path a: X → M)	34.14 (26.90, 41.38)	<0.001	84.73%
**Mediator effect on Gross motor** (Path b: M → Y)	−0.09 (−0.16, −0.02)	0.center	
**Indirect effect** (Path a^*^b: X → M)	−3.14 (−6.01, −0.20)	–	
**Direct effect** (Path c′: X → Y adjusted M)	−0.57 (−4.21, 3.08)	0.759	
**Total effect** (Path c: X → Y adjusted M)	−3.71 (−6.48, −0.93)	0.009	

ASQ-3, Age and Stage Questionnaire Chinese version; CI, confidence interval. X, independent variable (SARS-CoV-2 infection). M, mediator (mother–infant separation days). Y, dependent variable (score of gross motor development). Path a, the effect of SARS-CoV-2 infection on mediator. Path b, the effect of mediator on gross motor development. Path a^*^b (indirect effect), the mediated effect of SARS-CoV-2 infection on gross motor development by mother–infant separation days. Path c′ (direct effect), the remaining effect of SARS-CoV-2 infection on gross motor development extent not explained by the mediators included in the model. Path c (total effect), the total effect of SARS-CoV-2 infection on gross motor development. In the mediation model, we adjusted maternal education (bachelor or above vs. high school or below), low birthweight (yes vs. no), infant gender (boy vs. girl), preterm (yes vs. no), admitted to NICU (yes vs. no), and breastfeeding at 3 months after birth (yes vs. no).

## Discussion

This is the first cohort study to evaluate the potential direct and indirect effects of SARS-CoV-2 infection during pregnancy (mainly in the third trimester) on early childhood development. We followed 57 mother–infant dyads in an infection cohort and 78 dyads in a non-infection cohort until 3 months after delivery. Our findings showed that SARS-CoV-2 infection during pregnancy did not increase the risks of social–emotional (RR = 0.87, 95% CI: 0.51–1.49) and overall (RR = 1.02, 95% CI: 0.60–1.73) developmental delay. However, the length of mother–infant separation was significantly negatively correlated with the gross motor score (β = −0.09, 95% CI: −0.16 to −0.02). Furthermore, using a mediation model, we found that SARS-CoV-2 infection had a significant indirect effect on the gross motor score by increasing the length of mother–infant separation.

Betacoronaviruses, including SARS-CoV, MERS-CoV, and SARS-CoV-2, have caused severe global pandemics. A study after the 2003 SARS epidemic showed that SARS-CoV infection during pregnancy did not increase the risk of developmental delay in children compared with children of mothers who were not infected with SARS-CoV during pregnancy (19). There was no evidence that MERS-CoV infection during pregnancy affected early offspring development. Current evidence suggests that SARS-CoV, MERS-CoV, and SARS-CoV-2 affect fewer children, cause fewer symptoms, and less severe disease, and are associated with much lower case-fatality rates, compared with adults (20, 21). Studies have proposed a potential mechanism of SARS-CoV-2 infection during pregnancy on early offspring development, including maternal–fetal interface destruction, maternal systemic immune response, and direct brain infection interfering with brain development (22); however, these hypotheses have not been verified. Our findings provide epidemiological evidence that SARS-CoV-2 infection during pregnancy does not increase the risk of early developmental delay in offspring, compared with non-infected infants. In addition, it should be noted that the mothers in the infection cohort in the present study were mainly infected in the third trimester (accounting for 93.0%). Our results cannot explain the impact on the neurodevelopment of offspring when the infection occurs in the early pregnancy, especially in the first trimester, which needs to be explained by more studies in the future.

In the further mediation model, we found that SARS-CoV-2 infection during pregnancy had significant indirect effects on the ASQ-3 gross motor domain score by increasing the length of mother–infant separation. After separating the indirect effects, the remaining direct effect was not significant, which was consistent with the results of the multivariable linear regression models. To prevent neonatal infection (11), infants in the infection cohort experienced longer separation from their mothers than the non-infection cohort, which means that the infants in the infected group did not receive enough support and response from their mothers to meet their developmental needs (12). Early separation interrupted skin-to-skin contact, breastfeeding, and mother–infant interactions, which had adverse effects on the physiological development (23), innate and specific immunity (24), and neurodevelopment (including language, cognition, motor, and social development) of the children (25). In particular, we observed that the breastfeeding rate of the non-infected cohort at 1 and 3 months was 100%, which was significantly higher than that of the infection cohort. Better breastfeeding would be more conducive to early childhood development. Although this study did not find its significant effect, it still deserves continuous attention. Thus, we confirmed the hypothesis of the mediation model that mother–infant separation may have a potential impact on early childhood development. Furthermore, our results also indicated that although the impact of SARS-CoV-2 infection during late pregnancy on early childhood development is limited, the secondary effects of the COVID-19 pandemic (such as mother–infant separation) may still affect early childhood development. It is also worth noting that a published study showed that when adequate protective measures are taken, the risk of infant infection caused by rooming-in practice is limited (26). Combined with our results, it is necessary to focus on the benefit of protected rooming-in, on the premise that the risk of infant infection can be effectively controlled.

The COVID-19 pandemic has caused both a health crisis and economic and social crises (27–29), which have profoundly affected sustainable development goals (30). The mother–infant separation discussed here is only the tip of the iceberg of this global crisis. The World Bank Group estimates that the global economy contracted 4.3% in 2020; the pandemic raised poverty rates to between 9.1 and 9.4%, back to levels last seen in 2017 (31). Poverty is the source of many risk factors for early childhood development, including malnutrition, caregiver stress and depressive symptoms, low caregiver responsiveness, low-quality nurturing care, and community environment, causing them to miss early education (32). The COVID-19 pandemic is accelerating the deterioration of these external factors, pushing the poorest and most vulnerable people and their children into worse situations. We call for global attention to the plight of these families and their children in the current pandemic.

Our study had some limitations. First, the study population was small, since NERS reported only 138 pregnant women confirmed with COVID-19 as of April 30, 2020, which might affect the power of our study. Second, we found that only the length of mother–infant separation was negatively associated with the ASQ-3 gross motor domain score. Therefore, we used the gross motor domain score in the mediation model instead of the classification result. Therefore, we found only that SARS-CoV-2 infection had a potential indirect negative effect on the development of the children, which did not mean that this indirect effect would increase the risk of developmental delay in the offspring. In addition, the recruitment and follow-up were conducted through the internet and by telephone, and participant compliance was not as satisfactory as with face-to-face follow-up. Since our follow-up lasted only 3 months, our results cannot represent longer-term effects. Finally, we used screening scales instead of diagnostic scales to access early childhood development, which might cause evaluation bias.

In conclusion, we found that SARS-CoV-2 infection during late pregnancy did not increase the risk of offspring developmental delay at 3 months. However, we found that the length of mother–infant separation was significantly negatively correlated with the ASQ-3 gross motor domain score. We found that SARS-CoV-2 infection indirectly affected the gross motor score by increasing the length of mother–infant separation, mediating 84.73% of the total effect. Future researchers should conduct longer follow-up studies of children whose mothers were infected with SARS-CoV-2 during pregnancy, to evaluate the long-term effects of COVID-19 on early childhood development. Future researchers also need to pay more attention to potential mediators and indirect effects between COVID-19 and early childhood development as the pandemic is now changing the external environment of child development.

## Data Availability Statement

The raw data supporting the conclusions of this article will be made available by the authors, without undue reservation.

## Ethics Statement

The studies involving human participants were reviewed and approved by Peking University Third Hospital Medical Science Research Ethics Committee (No. IRB00006761–M2020127). The patients/participants provided their written informed consent to participate in this study.

## Author Contributions

JQ, XW, YZ, and YWe conceived and designed the study. TW, LC, and YWa conducted the statistical analyses and drafted the initial manuscript. TW, LC, YWa, HS, JN, XY, ML, CT, HJ, and DZ conducted the follow-up surveys. HS and XW contributed to the interpretation of the data. All authors reviewed and revised the article. All authors read the final manuscript and approved submission.

## Funding

This study was funded by the Chinese Academy of Engineering (2020-KYGG-01-06), the National Natural Science Foundation of China (72042013), the Peking University Health Science Center (BMU2020HKYZX001), and the National Key Research and Development Program of China (2016YFC1000307-2).

## Conflict of Interest

The authors declare that the research was conducted in the absence of any commercial or financial relationships that could be construed as a potential conflict of interest.

## Publisher's Note

All claims expressed in this article are solely those of the authors and do not necessarily represent those of their affiliated organizations, or those of the publisher, the editors and the reviewers. Any product that may be evaluated in this article, or claim that may be made by its manufacturer, is not guaranteed or endorsed by the publisher.

## Abbreviations

COVID-19: coronavirus disease 2019
SARS-CoV-2: severe acute respiratory syndrome coronavirus 2
NERS: National Epidemic Reporting System
RT-PCR: real-time reverse transcription polymerase chain reaction
ASQ-3: Age & Stage Questionnaire Chinese version
ASQ:SE-2: Age & Stage Questionnaire Social-Emotional Chinese version
IQR: interquartile range
RR: relative risk
NICU: neonatal intensive care unit
SARS-CoV: severe acute respiratory syndrome coronavirus
MERS-CoV: Middle East respiratory syndrome coronavirus.
